# Cluster analysis of resistance combinations in *Escherichia coli* from different human and animal populations in Germany 2014-2017

**DOI:** 10.1371/journal.pone.0244413

**Published:** 2021-01-20

**Authors:** Beneditta Suwono, Tim Eckmanns, Heike Kaspar, Roswitha Merle, Benedikt Zacher, Chris Kollas, Armin A. Weiser, Ines Noll, Marcel Feig, Bernd-Alois Tenhagen

**Affiliations:** 1 Department Biological Safety, Unit Epidemiology, Zoonoses and Antimicrobial Resistance, German Federal Institute for Risk Assessment, Berlin, Germany; 2 Department Infectious Disease Epidemiology, Unit Healthcare-associated Infections, Surveillance for Antibiotic Resistance and Consumption, Robert Koch Institute, Berlin, Germany; 3 Unit Antibiotic Resistance Monitoring, Federal Office of Consumer Protection and Food Safety, Berlin, Germany; 4 Department of Veterinary Medicine, Institute for Veterinary Epidemiology and Biostatistics, Working Group Applied Epidemiology, Free University Berlin, Berlin, Germany; Nitte University, INDIA

## Abstract

Recent findings on Antibiotic Resistance (AR) have brought renewed attention to the comparison of data on AR from human and animal sectors. This is however a major challenge since the data is not harmonized. This study performs a comparative analysis of data on resistance combinations in *Escherichia coli* (*E*. *coli*) from different routine surveillance and monitoring systems for human and different animal populations in Germany. Data on *E*. *coli* isolates were collected between 2014 and 2017 from human clinical isolates, non-clinical animal isolates from food-producing animals and food, and clinical animal isolates from food-producing and companion animals from national routine surveillance and monitoring for AR in Germany. Sixteen possible resistance combinations to four antibiotics—ampicillin, cefotaxime, ciprofloxacin and gentamicin–for these populations were used for hierarchical clustering (Euclidian and average distance). All analyses were performed with the software R 3.5.1 (Rstudio 1.1.442). Data of 333,496 *E*. *coli* isolates and forty-one different human and animal populations were included in the cluster analysis. Three main clusters were detected. Within these three clusters, all human populations (intensive care unit (ICU), general ward and outpatient care) showed similar relative frequencies of the resistance combinations and clustered together. They demonstrated similarities with clinical isolates from different animal populations and most isolates from pigs from both non-clinical and clinical isolates. Isolates from healthy poultry demonstrated similarities in relative frequencies of resistance combinations and clustered together. However, they clustered separately from the human isolates. All isolates from different animal populations with low relative frequencies of resistance combinations clustered together. They also clustered separately from the human populations. Cluster analysis has been able to demonstrate the linkage among human isolates and isolates from various animal populations based on the resistance combinations. Further analyses based on these findings might support a better one-health approach for AR in Germany.

## Introduction

Antibiotic resistance (AR) poses a global threat to humans, animals, and the environment [1]. AR in humans and different animal populations has increased in recent years. As noted in a recent report [2], European countries have dealt with 670,000 human infections with resistant bacteria in the year 2015. Third-generation cephalosphorin-resistant *Escherichia coli* (*E*. *coli*) were the major cause with 290,000 infections. In animals, *E*. *coli* has also been studied intensively in recent years since Extended-Spectrum-Beta-Lactamase/AmpC producing *E*. *coli* (ESBL/AmpC *E*. *coli*) have been detected in food-producing animals [3, 4]. *E*. *coli* have, however, not only been seen as very important pathogenic bacteria in humans and animals, but also as indicator bacteria or commensal bacteria in animals that may play a specific role in the transmission of AR genes from animals to humans [5, 6]. In order to tackle the increase in AR coming from numerous sectors, a multi-disciplinary approach is necessary, as humans, animals and the environment share similar resistance genes [7–12]. A “One Health” approach combines human, animal and environmental sectors in order to study, for example, transmission within and between the different reservoirs. “One Health”-based initiatives have been launched on national, European and global levels to act on the spread of AR [13–17]. In Germany, the National Action Plan on Antimicrobial Resistance (DART 2020, [18]) prioritizes adaption of this approach both nationally and internationally. One major challenge in adapting the “One Health” approach in Germany is the harmonization of data coming from various surveillance and monitoring systems on AR. First, in concordance with DART 2020, this study addresses the comparison of the various surveillance and monitoring systems on AR in human and veterinary medicine in Germany. Second, we describe resistance combinations in each population using phenotypic AR-data of non-clinical *E*. *coli* isolates from various food-producing animal populations including foods, clinical *E*. *coli* isolates from food-producing and companion animal populations and clinical *E*. *coli* isolates from different human populations collected through these surveillance and monitoring systems. *E*. *coli* is used as a model organism because of its prevalence in animals and humans, as well as the availability of respective data in Germany. In this study, non-clinical *E*. *coli* data from different food-producing animal populations and food defined the commensal *E*. *coli*. Finally, cluster analysis based on the relative frequencies of resistance combinations was used to study similarities in resistance combinations of *E*. *coli* isolates from the investigated populations.

## Materials and methods

### Ethic statements

For human datasets, this study has solely included anonymised routine surveillance data. Ethical approval for analysis of such surveillance data is not required according to the Medical Association’s professional code of conduct. Data on antimicrobial resistance of *E*. *coli* from animals and food were collected in the framework of national monitoring projects and have been published in aggregated form in the National reports as provided in the reference list. The data basis of this analysis is presented in S4 Table.

### Surveillance and monitoring of Antibiotic Resistance (AR) in Germany

*Antibiotika-Resistenz-Surveillance* (ARS) is the German national surveillance system for AR in humans. It is coordinated by the Robert Koch Institute (RKI) since 2007. The system collects routine laboratory data on AR in different bacterial pathogens that originate from clinical samples of patients in health care facilities (in- and outpatient care). It stores information on demographics (e.g. age and gender of the patients), type and region of health care facility as well type of hospital ward. Aggregated ARS datasets are sent to the European Antimicrobial Resistance Surveillance Network (EARS-Net) in the European Centre of Disease Prevention and Control (ECDC) and published annually. The participation of the laboratories in ARS is voluntary [19]. Seventeen commercial diagnostic laboratories covering 187 hospitals and 3,436 general practices have participated continuously in ARS from the year 2014 to 2017 (Status: May 2020). Antimicrobial susceptibility testing (AST) is conducted in the laboratories with routine diagnostic procedures, such as automated broth-microdilution (ISO standard 20776–1) [20] or agar disk diffusion [21]. Results are presented as susceptible (S), intermediate (I) and resistant (R) (SIR) based on internationally harmonized evaluation criteria such as clinical breakpoints provided by the European Committee on Antimicrobial Susceptibility Testing (EUCAST) and the Clinical Laboratory Standards Institute (CLSI).

*Zoonosis-Monitoring* (ZoMo) is the German monitoring system for AR in healthy food producing animals and food. It is a collaboration between federal institutions (German Federal Institute for Risk Assessment (BfR) and Federal Office of Consumer Protection and Food Safety (BVL)), regional veterinary and food safety authorities and regional public laboratories. *Zoonosis-Monitoring* has been implemented as national regulation according to Directive 2003/99/EC [22]. Details on mandatory bacteria-commodity combinations, antimicrobials used in the testing, laboratory methods and evaluation criteria for the determined minimum inhibitory concentrations (MIC) are fixed in Commission Implementing Decision (CID) 2013/652/EU [23]. In Germany, the federal states’ food safety authorities annually decide on a sampling plan. They collect representative samples at different levels (farm, slaughter, retail) of different food chains according to this sampling plan. Regional laboratories run by the federal states isolate the bacteria from the samples and submit them to the National Reference Laboratory for Antimicrobial Resistance (NRL-AR). AST at the NRL-AR is done according to CID 2013/652/EU using broth-microdilution. For *E*. *coli* there is a fixed panel of 14 antibiotics used in the testing (S1 Table). The MIC values are interpreted using Epidemiological Cut-Off (ECOFF) values published by EUCAST and laid down in the CID. Results are reported to the European Food Safety Authority (EFSA) and included in the annual “European Union summary report on antimicrobial resistance in zoonotic and indicator bacteria from humans, animals and food” [6]. At the national level they are reported annually by the BVL [24–27].

The *German Resistance Monitoring* (GE*RM*-Vet) on AR in animal pathogens is coordinated and conducted by the BVL. Based on §77 [3] of the German Medicinal Products Act (AMG), the BVL must report these data to the Federal Ministry of Food and Agriculture (BMEL) annually. Thirty-two participating public, private, and university laboratories submit voluntarily bacterial pathogens from diseased animals based on an annual sampling plan for different animal populations and indications. This annual sampling plan is established together with participating laboratories based on the experience from the previous years. Background information on the animals that has been sampled (e.g. age, disease) is also stored in the system. A customized BVL fixed panel of 24 antibiotics is used for AST in *E*. *coli* using broth microdilution (S1 Table). MIC values with CLSI breakpoints for animal pathogens are routinely reported [28–30]. Table 1 summarizes the comparison between the three German surveillance and monitoring systems.

**Table 1:** Comparison of surveillance and monitoring systems for AR in humans and animals in Germany.

Variable	ARS	ZoMo	GE*RM*-Vet
Type of bacteria	Human clinical isolates	Animal non-clinical isolates (commensal and food)	Animal clinical isolates
Participation	Voluntary	Mandatory	Voluntary
Population	Humans	Animal species and food	Animal species
AST panel	Not harmonized	Harmonized Panel	Harmonized Panel
		14 substances	24 substances
AST methods	Broth-Microdilution	Broth—Microdilution	Broth—Microdilution
	(kinetic growth curves)		
AST results	‘susceptible’, ‘intermediary’, ‘resistant’ (SIR) or MIC	Minimum Inhibitory Concentration (MIC)	Minimum Inhibitory Concentration (MIC)
Evaluation criteria	EUCAST / CLSI clinical breakpoints	EUCAST-ECOFFs	CLSI clinical breakpoints for animals
Accreditation	All laboratories	All laboratories	All laboratories

*AST: antimicrobial susceptibility testing.

### Description of data and study design

We included *E*. *coli* data available in ARS, ZoMo and GE*RM*-Vet from January 2014 to December 2017. From ARS we took only data from laboratories and health care facilities in Germany, which participated in the system continuously from January 2014 to December 2017. The first isolate per patient per type of clinical specimen per year was used for the analysis. Screening samples, duplicate isolates (same type of clinical specimen from the same patient) and isolates with incomplete information were excluded. All used types of specimen are listed in Tables 2 and S3. This study focused on qualitative interpretation of AST (SIR) according to EUCAST clinical breakpoints. Further, ARS-data were classified by type of health care facility, i.e. human isolates from intensive care units (ICU), general wards and from outpatient care. We included all *E*. *coli* isolates from the annual sampling plans in ZoMo between 2014 and 2017. A summary of these data has been previously published in annual national reports [24–27]. ZoMo data include food-producing animals’ isolates from farms, slaughterhouses and from food at retail from all German federal states (Table 2). Distribution of the samples across the federal states was proportionate to the number of animals of the targeted animal population in the federal state for samples taken on farms. For slaughterhouse samples, the distribution was proportionate to the slaughter capacity within the federal state for the targeted animal population. Numbers of samples at retail were based on the distribution of the human population. All materials are listed in Table 2.

**Table 2:** *Escherichia coli* data for different populations collected from *Zoonosis-Monitoring*, GE*RM*-Vet and ARS from 2014 to 2017.

Isolate type	Origin	Populations	Materials	Year			
				2014	2015	2016	2017
**Non-clinical animal isolates** (27 populations, incl 9 food)	Farm (F)	Broilers, F	Faeces			X	
		Broilers Conv, F	Faeces			X	
		Broilers Org, F	Faeces			X	
		Laying hens, F	Faeces	X			
		Breeder chickens, F	Faeces	X			
		Turkeys, F	Faeces	X		X	
		Growers <50 kg, F	Faeces				X
		Weaners, F	Faeces from waiting area		X		
		Sows, F	Faeces of pregnant sows		X		
		Bovine milk, Conv, F	Bulk tank milk	X			
		Bovine milk, Org, F	Bulk tank milk	X			
		Bivalves, F	Both of shells meat			X	
	Slaughter (S)	Broilers, S	Pool from ten caecals	X		X	
		Turkeys, S	Pool from ten caecals	X		X	
		Bovines <1year, S	Caecals		X		X
		Fattening pigs, S	Caecals		X		X
	Retail (R)	Venisons, R	Fresh Meat				X
		Shrimps, R	Shrimps Meat		X		
		Broiler meat, R	Fresh meat with skin	X		X	
		Table eggs, R	Pool from ten eggshells	X			
		Turkey meat, R	Fresh meat with skin	X		X	
		Bovine meat, R	Fresh meat		X		
		Pork, R	Fresh meat		X		X
		Raw sausages, R	Fresh meat				X
		Bivalves, R	Both of shells meat	X		X	
	Wild/Game	Roe deer hunted, W	Faeces				X
		Wild boar hunted, W	Faeces			X	
**Clinical animal isolates (C)** (11 populations)	Farm/veterinary practice	Piglets, C	Faeces / Intestines/ Swab (Enteritis)	X	X	X	X
		Growers, C	Faeces / Intestines/ Swab (Enteritis)	X	X	X	X
		Pigs, C	Faeces / Intestines/ Swab (Enteritis)	X	X	X	X
		Sows, C	Not specified^+^ (Mastitis-Metritis-Agalactie—MMA)	X	X	X	X
		Broilers, C	Not specified^+^ (Septicemia)	X	X	X	X
		Laying hens, C	Not specified^+^ (Septicemia)	X	X	X	X
		Turkeys, C	Not specified^+^ (Septicemia)	X	X	X	X
		Bovines <1year, C	Faeces / Intestines/ Swab (Enteritis)	X	X	X	X
		Cattle, C	Faeces / Intestines/ Swab (Enteritis)	X	X	X	X
		Dairy cows, C	Not specified^+^ (Mastitis)	X	X	X	X
		Small animals, C	Not specified^+^ (Enteritis/Urinary Tract Infection)	X	X	X	X
**Clinical human isolates** (3 populations)	Outpatient	Humans, A	All kind of swabs, blood, punctate, respiratory tract samples, wound samples, urine and other samples***	X	X	X	X
	General Ward	Humans, Gw		X	X	X	X
	Intensive care unit (ICU)	Humans, ICU		X	X	X	X

^+^Clinical specimens are not specified, only disease information was obtained.

*Data collected from conventional (conv) and organic (org) farms.

**Small animals are cats and dogs.

***All details of materials are listed in S3 Table.

Materials indicate where the specimen that the isolates originated from. Year indicates the different sampling year plan for the non-clinical and clinical animal isolates and the food isolates.

The GE*RM*-Vet study year lasts from April to March from each observation year. In this study we included all *E*. *coli*-isolates, which had been collected from January 2014 to December 2017 (study years 2013 to 2017). A summary of the data has been published previously in annual reports [28–30]. The isolates originated from diseased animals, which had not been treated with antibiotics in the month prior to sampling. All materials along with the information on diseases are listed in Table 2.

Four antibiotics were selected for the cluster analysis: ampicillin (AMP), cefotaxime (CTX), ciprofloxacin (CIP) and gentamicin (GEN). They are included in the test panels of ZoMo and GE*RM*-Vet and likewise frequently tested in the medical laboratories reporting to ARS. Other relevant antibiotics for *E*. *coli* such as colistin, carbapenems, co-trimoxazol, tetracycline could not be included in this study because of the limited data available in the different systems. This will be further explained in the discussion section. All isolates from ARS, which had not been tested against all of these four antibiotics, were excluded from the analysis. EUCAST clinical breakpoints for human clinical isolates (S2 Table) were used to interpret the MIC-values from animal and food isolate data.

### Statistical analysis

All MIC values were coded as 0 for susceptible and 1 for resistant. Intermediate results of human AST were interpreted as susceptible. Once the coding was complete, the relative frequency of all 16 possible combinations of resistance to the four antimicrobials was calculated for each population using the permutation function (2^4^ = 16). Resistance proportions were calculated using number of tested isolates for each population as denominator. The relative frequency of the resistance combinations (in %) was determined for each population. Building on Jasper et al. [31], we modified hierarchical clustering based on relative frequencies of the resistance combinations for phenotypical AR data. We did not use the suggested principal component analysis (PCA) for choosing the resistance combinations, since we had only four antibiotics included. We tested hierarchical clustering using numerous distance measures: single (nearest neighbor), complete (furthest neighbor), and average linkage (average between nearest and furthest neighbor) and Ward’s method [32]. However, average linkage with Euclidean distance was selected since it produced the most meaningful results. A dendrogram and a heatmap were used to visualize the results. In addition to cluster descriptions based on the visualization in a dendogram, we used the elbow method and silhouette plot [33] for confirming the number of clusters. All analyses were run with R 3.5.1 (Rstudio 1.1.442).

### Sensitivity analysis

In an attempt to test the robustness of the result we performed sensitivity analyses. We carried out four analyses, during which one antibiotic at a time was removed from the data. Thus, the total number of antibiotics in these reduced models was three, resulting in eight different resistance combinations each. Then, we used our clustering approach to further analyze the reduced models. Results were compared to clustering using all four antibiotics (complete model).

## Results

### Description of included isolates

**Fig 1:**
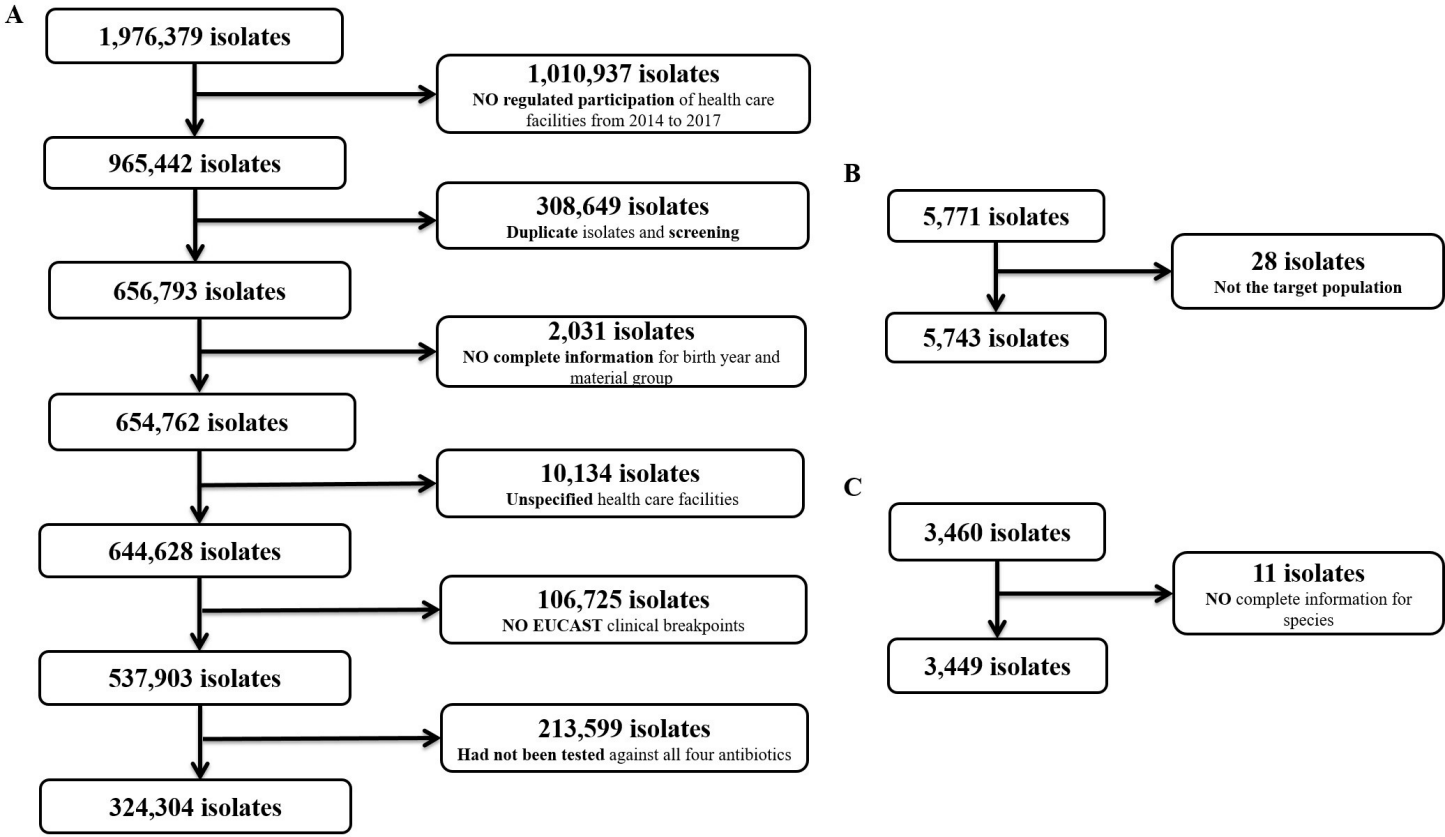
Data extraction from three surveillance and monitoring systems for AR. A) ARS system; B) Zoonosis-Monitoring and C) GE*RM*-Vet.

333,496 *E*. *coli* isolates were included from ARS, ZoMo and GE*RM*-Vet between January 2014 and December 2017. 324,304 isolates (97.2%) originated from human populations, 5,743 isolates (1.7%) from healthy food-producing animals and food and 3,449 isolates (1.0%) from diseased animals. Extraction of the data for each surveillance and monitoring system is described in Fig 1A–1C. Most human isolates (210,005 isolates (64.8%)) originated from urine samples (S3 Table). Forty-one different populations were defined including 3 human populations, 18 healthy food producing animal populations, 9 food items and 11 diseased animal populations contributing clinical *E*. *coli* isolates (Table 2).

### Resistance to the four antimicrobials in isolates from the different populations

Table 3 demonstrates individual resistance proportions of *E*. *coli* from the different human and animal and food populations to each antibiotic. Overall, resistance proportions were highest to ampicillin, followed by ciprofloxacin, cefotaxime and gentamicin. They ranged from 43% to 55% in human clinical isolates, from 1% to 70% in healthy food-producing animals including wild animals (game) and food and from 16% to 64% in clinical animal isolates. Human clinical isolates from ICU, isolates from several healthy poultry populations (broilers and turkeys from farm, and slaughterhouse and their meats at retail), and clinical isolates from bovines <1 year showed the highest resistance proportions to all included antibiotics.

**Table 3:** Individual resistance proportions (%) from different populations against four selected antibiotics; ampicillin (AMP), cefotaxime (CTX), ciprofloxacin (CIP) and gentamicin (GEN).

Isolate Type	Origin	Populations	Number of tested isolates ^a^ (N)	Resistance Proportions [95% CI] (%)			
				AMP	CTX	CIP	GEN
**Non-clinical animal isolates** (27 populations, incl 9 food)	Farm (F)	Weaners, F	250	50.8 [44.4; 57.2]	4.4 [2.2; 7.7]	4.0 [1.9; 7.2]	2.4 [0.9; 5.2]
		Laying hens, F	347	13.3 [9.9; 17.3]	3.2 [1.6; 5.6]	2.0 [0.8; 4.1]	0.9 [0.2; 2.5]
		Broilers, F	184	72.3 [65.2; 78.6]	2.2 [0.6; 5.5]	22.8 [17.0; 29.6]	1.1 [0.1; 3.9]
		Broilers Conv, F	299	70.2 [64.7; 75.4]	1.0 [0.2; 2.9]	18.7 [14.5; 23.6]	1.0 [0.2; 2.9]
		Broilers Org, F	31	22.6 [9.6; 41.1]	0 [0; 11.2]	9.7 [2.0; 25.8]	3.2 [0; 16.7]
		Turkeys, F	346	62.4 [57.1; 67.6]	0.6 [0.1; 2.1]	23.1 [18.8; 28.0]	8.4 [5.7; 11.8]
		Growers <50 kg, F	210	34.8 [28.3; 41.6]	1 [0.1; 3.4]	2.4 [0.8; 5.5]	1.9 [0.5; 4.8]
		Bovine milk, Conv, F	122	5.7 [2.3; 11.5]	0 [0; 3]	0.8 [0; 4.5]	0 [0; 3]
		Bovine milk, Org, F	74	1.4 [0; 7.3]	0 [0; 5.0]	0 [0; 5.0]	0 [0; 5.0]
		Breeder chickens, F	56	25.0 [14.4; 38.4]	0 [0; 6.4]	7.1 [2.0; 17.3]	5.4 [1.1; 14.9]
		Sows, F	24	26.5 [21.3; 32.1]	1.5 [0.4; 3.7]	1.8 [0.6; 4.2]	2.2 [0.8; 4.7]
		Bivalves, F	42	9.5 [2.7; 22.6]	0 [0; 8.4]	0 [0; 8.4]	2.4 [0; 12.6]
	Slaughter (S)	Broilers, S	404	57.2 [52.2; 62.1]	0.3 [0.1; 1.4]	10.6 [7.8; 14.1]	6.4 [4.3; 9.3]
		Bovines <1year, S	433	34.2 [29.7; 38.9]	1.8 [0.8; 3.6]	2.8 [1.4; 4.8]	0.9 [0.3; 2.3]
		Turkeys, S	372	63.7 [58.6; 68.6]	1.6 [0.6; 3.5]	19.6 [15.7; 24.0]	8.1 [5.5; 11.3]
		Fattening pigs, S	439	31.2 [26.9; 35.8]	2.5 [1.3; 4.4]	2.1 [0.9; 3.9]	0.5 [0; 1.6]
	Retail (R)	Venisons, R	150	2 [0.4; 5.7]	0 [0; 2.4]	0 [0; 2.4]	0 [0; 2.4]
		Shrimps, R	20	20 [5.7; 43.7]	10 [1.2; 31.7]	10 [1.2; 31.7]	5 [0; 24.9]
		Broiler meat, R	363	54.8 [49.5; 60.0]	4.4 [2.5; 7.1]	19.0 [15.1; 23.4]	3.0 [1.5; 5.4]
		Table eggs, R	90	11.1 [5.5; 19.5]	0 [0; 4]	1.1 [0; 6.0]	1.1 [0; 6.0]
		Turkey meat, R	356	67.4 [62.3; 72.3]	3.4 [1.8; 5.8]	21.3 [17.2; 26.0]	8.7 [6.0; 12.1]
		Bovine meat, R	115	11.3 [6.2; 18.6]	2.6 [0.5; 7.4]	0.9 [0; 4.8]	0 [0; 3.2]
		Pork, R	155	25.2 [18.5; 32.8]	2.6 [0.7; 6.5]	0 [0; 2.3]	0.6 [0; 3.5]
		Raw sausage, R	69	20.3 [11.6; 31.7]	1.5 [0; 7.8]	7.3 [2.4; 16.1]	1.5 [0; 7.8]
		Bivalves, R	58	8.6 [2.9; 19.0]	0 [0; 6.1]	3.5 [0.4; 11.9]	0 [0; 6.1]
	Wild/Game (W)	Roe deer hunted, W	269	1.5 [0.4; 3.8]	0.4 [0.1; 2.1]	0 [0; 1.4]	0 [0; 1.4]
		Wild boar hunted, W	217	0.5 [0; 2.5]	0 [0; 1.7]	0.9 [0.1; 3.3]	0 [0; 1.7]
**Clinical animal isolates (C)** (11 populations)	Farm/veterinary practice	Piglets, C	417	61.4 [56.5; 66.1]	6.5 [4.3; 9.3]	8.9 [6.3; 12.0]	7.2 [5.0; 10.1]
		Laying hens, C	557	15.6 [12.7; 18.9]	0.9 [0.3; 2.1]	2.3 [1.3; 4.0]	1.8 [0.9; 3.3]
		Bovines <1year, C	534	71.4 [67.3; 75.2]	30.5 [26.6; 34.6]	36.0 [31.9; 40.2]	29.4 [25.6; 33.5]
		Small animals, C	312	34.3 [29.0; 39.9]	10.3 [7.1; 14.2]	16.4 [12.4; 20.9]	5.8 [3.5; 9.0]
		Growers, C	129	63.6 [54.6; 71.9]	7.0 [3.2; 1.3]	2.3 [0.5; 6.6]	4.7 [1.7; 9.8]
		Broilers, C	232	35.4 [29.2; 41.9]	3.9 [1.8; 7.2]	5.2 [2.7; 8.9]	4.7 [2.4; 8.3]
		Dairy cows, C	378	18.5 [14.7; 22.8]	7.1 [4.8; 10.2]	6.1 [3.9; 9.0]	3.4 [1.8; 5.8]
		Turkeys, C	327	40.4 [35.0; 45.9]	0.3 [0; 1.7]	5.5 [3.3; 8.6]	4.3 [2.4; 7.1]
		Cattle, C	193	47.2 [39.9; 54.5]	14.5 [9.9; 20.3]	22.8 [17.1; 29.4]	15.0 [10.3; 20.9]
		Pigs, C	346	49.7 [44.3; 55.1]	5.2 [3.1; 8.1]	6.1 [3.8; 9.1]	4.6 [2.7; 7.4]
		Sows, C	24	29.2 [12.6; 51.1]	0 [0; 14.2]	4.2 [0.1; 21.1]	4.2 [0.1; 21.1]
**Clinical human isolates** (3 populations)	Outpatient (A)	Humans, A	96,455	42.7 [42.4; 42.9]	7.3 [7.2; 7.4]	15.2 [15.0; 15.3]	4.8 [4.7; 4.9]
	General Ward (Gw)	Humans, Gw	197,521	49.2 [49.2; 49.4]	11.5 [11.3; 11.6]	19.5 [19.4; 19.6]	5.8 [5.7; 5.9]
	Intensive Care Unit (ICU)	Humans, ICU	30,328	54.9 [54.4; 55.5]	15.8 [15.4; 16.1]	22.0 [21.5; 22.4]	6.9 [6.6; 7.2]

^**a**^Number of tested isolates is the sum of all sensible (0) and all resistant (1) isolates.

The denominator was number of isolates from the respective population tested against each antibiotic from 2014 to 2017.

### Cluster analysis and overall relative frequencies of resistance combinations in the populations

**Fig 2:**
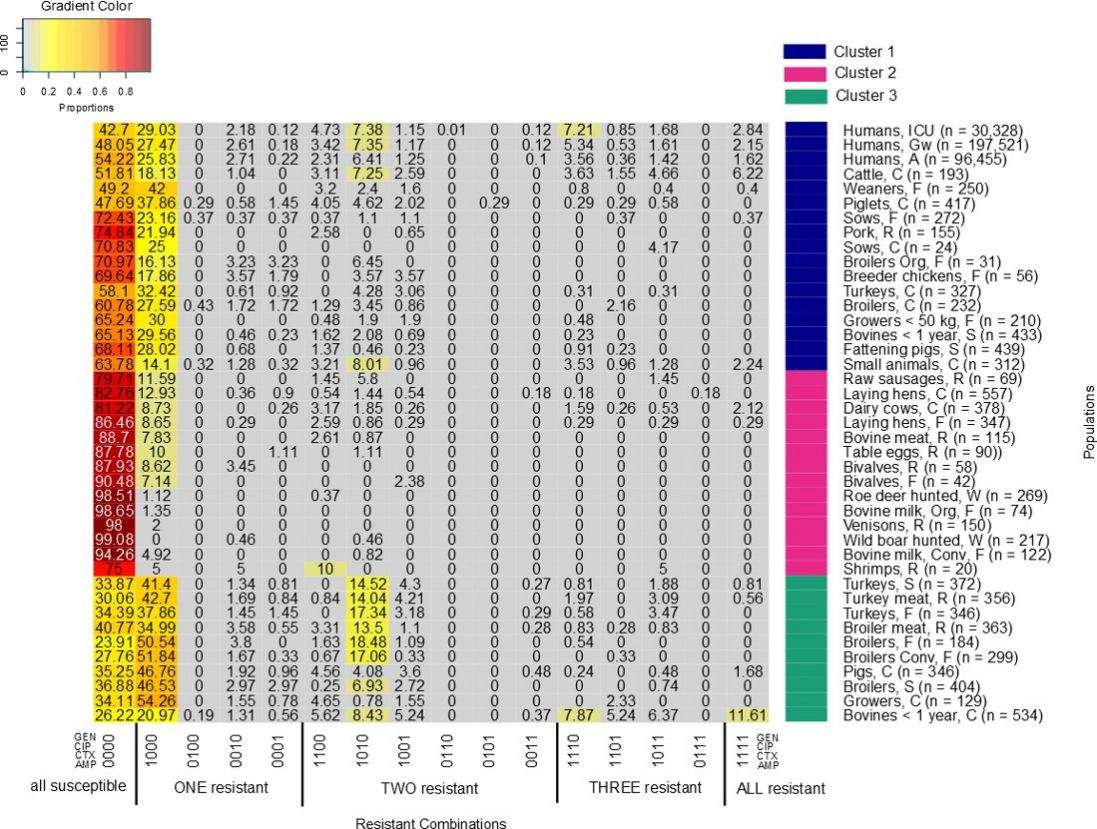
Heatmap describes different relative frequencies of resistance combinations for each population. From grey to red it shows the lowest to highest relative frequencies of resistance combinations. The x-axis explains the resistant combinations; from all susceptible (left) to all resistant (right). Antibiotics are described with ‘0’ as ‘susceptible’ and ‘1’ ‘resistant’. The order is GEN (gentamicin), CIP (ciprofloxacin), CTX (cefotaxime) and AMP (ampicillin). The y-axis denotes each population together with their clusters.

**Fig 3:**
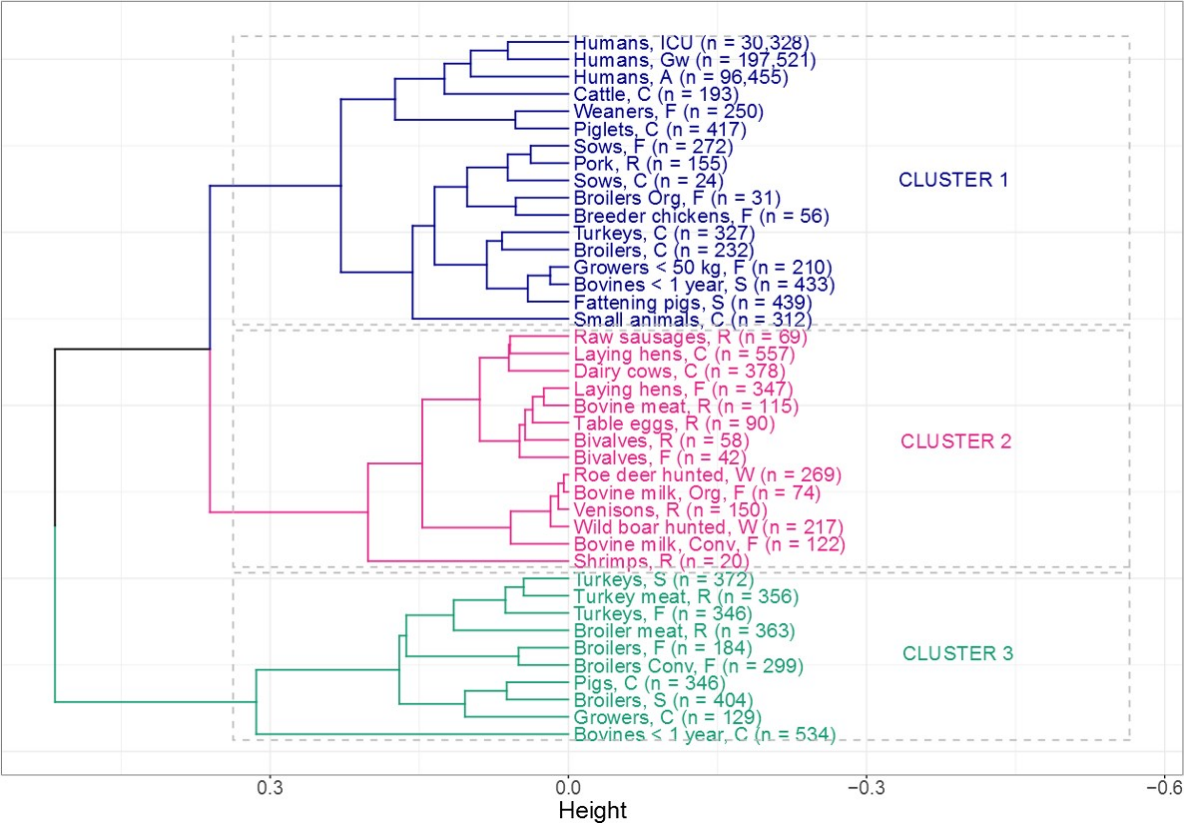
Cluster dendogram of different animal and human populations based on the relative frequency of resistance combinations to ampicillin, cefotaxime, ciprofloxacin and gentamicin. The x-axis describes the averaged similarities between the populations and between clusters. The y-axis shows each population and different clusters.

Three clusters were detected within our dataset (Figs 2 and 3) by visualizing the dendogram and confirming with the elbow method and silhouette plot (S1 Fig). The heatmap (Fig 2) highlights 16 resistance combinations; starting from “susceptible to all” to “resistant to all” (left to the right). Each column represents the relative frequency of a resistance combination for each population. Human isolates were mostly exclusively resistant to ampicillin (26–29%), followed by resistance to ampicillin and ciprofloxacin (6–7%), and resistance to ampicillin, cefotaxime and ciprofloxacin (4–7%). Isolates from most healthy broiler and turkey populations reported higher resistance proportions to ampicillin only (46–50%) and to ampicillin and ciprofloxacin (14–19%) compared to most other populations.

Human isolates of all three populations clustered closely together in the first cluster (Figs 2 and 3). The isolates from the three human populations had similar relative frequencies of resistance combinations. The cluster also included isolates from 14 animal/food populations in two sub-clusters. Six of these were clinical isolates including subpopulations of all major food producing animal species (i.e. cattle, pigs, broilers and turkeys) and companion animals. Clinical isolates from cattle and piglets and non-clinical isolates from weaned piglets clustered closest to the human isolates. Two of the healthy poultry populations (broilers from organic farms and breeder chicken) are included in this cluster. They are separated from other healthy poultry populations in cluster three. The second cluster mainly included populations, which had low relative frequencies of resistance combinations (<25%) for all tested antibiotics and high proportions of isolates that were susceptible to all tested antimicrobials. The cluster mostly included food at retail, wild animals, laying hens, and bulk tank milk from dairy herds including from organic dairy production. The third cluster contained mostly healthy poultry (7 out of the 10 populations). Besides healthy poultry the cluster contained isolates from poultry meat, clinical isolates from pigs and, slightly separated, clinical isolates from young cattle.

### Sensitivity analysis

Elimination of individual antibiotics from the model led to changes in the clusters (S2A–S2D Fig). In each elimination process, human inpatient isolates (ICU and general ward) always clustered together. Likewise, the sub-clustering of isolates from some of poultry isolates (broilers from conventional farms and all isolates from turkeys (farm, slaughterhouse and meat)); wild animals (roe deer hunted, wild boar hunted and venisons); bovine milk from organic and conventional farms remained in same sub-cluster.

After eliminating ampicillin data, most healthy poultry isolates (except broilers from organic farms, breeder chickens and laying hens from farms and broilers from the slaughterhouse) clustered together with human isolates. Human isolates from outpatient care clustered closely with clinical isolates from small animals instead of with clinical isolates from cattle but remained the next neighbor to the inpatient isolates (S2A Fig).

After eliminating cefotaxime data, human isolates from ICU and general ward likewise clustered with most of healthy poultry populations, again with the exception of broilers from organic farms, breeder chickens and laying hens from farms. In this model, we found that human isolates from ICU and general ward cluster closely with broiler meat at retail. Without considering cefotaxime, human isolates from outpatient care clustered separately from those of inpatients indicating that resistance to cefotaxime might be important for their close association in the full model. As in the full model, they clustered with clinical isolates from cattle.

Eliminating ciprofloxacin data, human isolates from ICU and general ward clustered with isolates from weaners, clinical isolates from piglets and broiler meat at retail. Human isolates from outpatient care stayed in one cluster with human isolates from ICU and general ward but not as their closest neighbor. They clustered together with clinical isolates from turkeys. All healthy poultry populations, again except broilers from organic farms, breeder chickens and laying hens from farms, clustered separately.

By eliminating gentamicin, the model outcome did not differ substantially from the complete model.

## Discussion

Cluster analysis provided information on similarities of *E*. *coli* isolates from humans and different animal populations based on their resistance combinations.

Human isolates from ICU and general ward always clustered together in cluster 1. Isolates from outpatient care were the next closest link in the full model and in two of the four reduced models. This finding supports the hypotheses that most ICU isolates are related to isolates from other parts of the hospital and from outpatients [34]. Studies on transmission within health-care-network and patient transfers have also supported this idea [35–37]. The slightly larger distance of the outpatient populations in comparison to the inpatient populations (general ward and ICU) might be explained by the specific situation in hospitals, with dominant hospital strains that differs from the outpatient setting [8]. Moreover, in the full model, isolates from the three human clinical populations clustered with clinical isolates from most (6/11) animal populations; i.e. cattle, piglets, sows, turkeys, broilers and small animals. The reason for these similarities between clinical isolates from human and different animal populations remains unclear as transmission of clinical isolates from animals to humans by contact or food is unlikely.

Isolates from most pig populations clustered together with the human clinical isolates (Cluster 1). This included clinical and non-clinical isolates from pigs. Prevalence of AR in pigs is associated with overall country-specific antimicrobial usage in livestock [38] Penicillins and tetracyclines are among the most frequently used antibiotics in pigs in Germany [39, 40]. This might explain the high proportions of resistance only to ampicillin in our study. Tetracyclines had to be excluded from this study as they were only tested in few medical laboratories. Their inclusion would have been associated with a substantial loss of data on the medical side as only isolates tested against all study antimicrobials could be included. The highest proportion of ampicillin-resistance in this cluster was found in weaners (fattening piglets, up to 30kg body weight) from farms (42%) (Fig 2). Higher single ampicillin resistance in weaners in comparison to other pig populations may have been caused by a higher treatment frequency with penicillins in piglets around and weaning time to address streptococcal infections in comparison to older fattening pigs [39, 40]. Two groups of clinical isolates from pigs and growers clustered separately from the other pig populations in cluster 3. This separation was associated with higher proportions of resistance only to ampicillin in these two populations and lower proportions of susceptibility to all four studied antimicrobials than in the other pig populations.

As for healthy pigs, the transmission of bacteria from pigs to humans could be explained via food consumption. Pork is occasionally consumed raw in Germany. It is in line with our study findings, which found isolates from pork and human clinical isolates in the same cluster. However, the clinical isolates are not likely to be transmitted via food as food is harvested from healthy animals. Another possible explanation is the similar antimicrobial usage (AMU)-pattern between humans and pigs for the antimicrobials included which may create similar resistance patterns, as penicillins are also frequently used in humans. In that case, the clustering would have been caused by parallel developments rather than by transmission of isolates. This explanation could also embrace the clinical isolates.

Our study indicates separate clusters for clinical human isolates (cluster 1) and isolates from most healthy broilers (except broilers from organic farms), and turkey populations and their meat (cluster 3) and for laying hens (cluster 2). It has been reported that extended-spectrum cephalosporin-resistant *E*. *coli* from healthy poultry are unlikely to be the causative agents of human UTI [41]. Another study revealed low similarities of ESBL/AmpC genes between broilers and the general human population with the exception of the broiler farming communities [8] In line with that, our study indicates a lack of similarities in resistance to the four antimicrobials of *E*. *coli* from human and healthy broiler and turkey populations and laying hens.

In the third cluster, healthy broilers and turkeys along with their meats clustered together. AR in non-clinical *E*. *coli* isolates from broilers is associated with antimicrobial use in poultry production. Resistance proportions in *E*. *coli* to penicillins and fluoroquinolones are reported to be 40% higher in countries which have allowed the use of these two antibiotics in poultry than countries which have not [42]. In Germany, ampicillin and enrofloxacin, a fluoroquinolone with a similar chemical structure as ciprofloxacin, are authorized antibiotics for the treatment of poultry [43]. The total treatment frequencies of penicillins and fluoroquinolones in fattening turkeys and chickens are higher compared to pigs and cattle [39]. This might be the reason for higher individual resistance proportions against ampicillin and ciprofloxacin and the higher relative frequencies of the combinations of resistance to both substances compared to other populations [43].

Three non-clinical poultry populations: broilers from organic farms, laying hens and breeder chickens, and two clinical poultry populations: broilers and turkeys were not included in this third cluster (Fig 3). Broilers from organic farms, laying hens and breeder chickens have lower individual resistance proportions against the studied antimicrobials compared to the other healthy poultry populations. This is in line with earlier work on lower resistance proportions in broilers and turkeys from organic farms [44–46]. Lower antibiotic resistance rates might be caused by lower antibiotic usage in organic farming. EU legislation governing organic farming (Reg. (EC) No. 834/2007) foresees the use of antibiotics solely for diseased animals, if phytotherapeutic drugs, homeopathy and other products are not working. This includes the restriction on number of treatments and longer duration of withdrawal periods [47, 48]. This may contribute to a lower use of antibiotics in organic broiler farming compared to conventional farming. However, valid specific use data from organic poultry farms are not available for Germany.

For breeder chickens and laying hens, low relative frequencies of resistance combinations were detected with resistance in laying hens even lower than in breeder chickens. Low single resistant proportions to the four chosen antibiotics in these two populations have been previously reported [49, 50]. Laying hens and breeder chickens received less antibiotic treatment than broilers, with the lowest antibiotic treatment in laying hens [51]. We, therefore, assume that the low relative frequencies of resistance combinations are associated with less antibiotic treatments received in laying hens and breeder chickens compared to broilers. Breeder chickens, i.e. parents and grand-parent flocks of production chicken, and laying hens live longer than broilers that only have a lifespan of approximately 4–6 weeks. It seems reasonable that the microbiome of breeder chickens and laying hens has matured [52, 53]. These microbiomes may be more competitive and resilient than those in young broilers contributing to less disease and therefore fewer treatments. Moreover, the housing conditions of breeder chickens are strictly controlled [54]. A controlled housing management might reduce the prevalence of pathogens and their transmission, which also results in fewer antibiotic treatments.

Clinical isolates from broilers and turkeys have lower resistance proportions to ampicillin compared to non-clinical isolates from broilers and turkeys (Fig 2). This applies also for the combined resistance proportions to ampicillin and ciprofloxacin. The reasons for these lower resistance rates in clinical isolates are however unclear and should be further investigated.

Isolates from wild animals, i.e. wild boars, wild roe deer and venison, clustered closely together with bulk tank milk both from conventional and organic farms. Isolates from these five populations showed the lowest individual resistance proportions and relative frequency of resistance combinations of all populations. Wild animals receive no antibiotic treatment, and therefore are not directly exposed to antimicrobials. However, wild animals were reported to carry AR commensal *E*. *coli* (non-clinical *E*. *coli* isolates) and play a role as sentinels of environmental transmission of AR [55, 56]. The presence of AR in wild animals has been associated to geographical distance to AR sources, such as wastes of antibiotic treated animals or humans [55], and also to human population density [57].

*E*. *coli* from bulk tank milk from both conventional and organic farms had low resistance rates and relative frequencies of resistance combinations. Low presence of AR in commensal *E*. *coli* (non-clinical *E*. *coli* isolates) from bulk tank milk has been previously reported [58, 59]. Low use of antibiotics in dairy cattle [51, 60] might result in low AR in the bacteria in milk. However, as *E*. *coli* is not part of the healthy milk microbiota and milk from *E*. *coli* mastitis is as a rule discarded, the most common source of *E*. *coli* in bulk tank milk is environmental, i.e. fecal contamination, mostly originating from the dairy herd [61] Improper milking-system hygiene also plays a role in milk contamination with coliform bacteria from the environment [62], but probably has no impact on their resistance patterns.

Clinical isolates from bovines <1 year had the highest individual proportions of AR for all four antibiotics as well as the highest relative frequency of the resistance combinations (Table 3 and Fig 2). This resulted in higher proportions of resistance combinations in comparison to other populations. Many of the isolates originated from young calves with enteritis. Use of waste milk may have contributed to the high resistance rates [63–65], given that penicillins and cephalosporins are frequently used in the treatment of mastitis of dairy cows [66, 67]. Waste milk is likely to contain residues of antimicrobials especially after intramammary treatment of dairy cows. This however cannot explain the comparatively high resistance rates to gentamicin and ciprofloxacin, as these substances are not frequently used in intramammary treatment. Further research into the dynamics of AR in calves is needed to improve the understanding of our study results.

Clinical animal isolates frequently clustered separately from their healthy animal counterparts. Our animal samples originated from two different independent datasets. There is no information whether they originated from the same farms. However, given the large number of farms and the limited number of isolates a large overlap of the source is unlikely. The separation might be caused by differences in selection pressure between the clinical and non-clinical isolates, although they originated from the same animal species and type of population. Non-clinical food-producing animal incl. food isolates were randomly sampled from each federal state in Germany. Clinical food-producing and companion animal isolates might be particular isolates from ill animals that form a specific subpopulation of *E*. *coli* strains. The GE*RM*-Vet study protocol states that the animals of origin should not have been treated with antibiotics within a month prior to sampling. However, it seems possible that these pathogenic isolates had prior specific antibiotic selection pressure in the animal population before the sampling time. An earlier study found the same tetracycline and aminoglycosides resistance genes in commensal (non-clinical isolates) and clinical *E*. *coli* [68]. Further research into the two different bacterial populations is necessary to better understand the reasons for the differences in AR.

With the sensitivity analysis we aimed to look into consistency of clusters built from the complete model (Fig 3). Some populations, i.e. human isolates from inpatient care (ICU and general ward) and isolates from wild animals and bovine milk from organic farm; remained in the same sub clusters consistently. This underlines their very close similarity with respect to resistance to the four antimicrobials and a distance to isolates from the other populations.

Removal of individual antimicrobials from the analysis also resulted in changes in cluster distributions compared to the complete model. The removal of one of the three antimicrobials—ampicillin, cefotaxime and ciprofloxacin—at a time made human clinical isolates from outpatient care change their position and nearest neighbors. This indicates a certain distance to the inpatient isolates. On the other hand, the change in the closest neighbor depending on the antimicrobial that was removed indicates that there was no clear relation to any individual other population. Removal of one antibiotic influenced the relative frequency proportions of resistance combinations. Resistance rates to ampicillin and ciprofloxacin were high in our study populations. Therefore, the removal of these two antibiotics substantially influenced the cluster order. In contrast, removal of gentamicin did not influence the clusters much. While a full analysis of these findings is outside the scope of this paper, we propose further analyses including additional antibiotics in order to understand the importance of different antibiotic usages in human and animal sectors.

There are a number of limitations to this study that must be acknowledged. Due to differences in the antimicrobials tested in the three systems, we had to choose four common antibiotics that overlapped between the three systems and for which sufficient data were available in ARS. Inclusion of further antimicrobials (e.g. tetracycline), would have reduced the number of available isolates in ARS substantially and would have excluded data from several laboratories, as those did not test *E*. *coli* for tetracycline resistance routinely. In ZoMo trimethoprim and sulfonamides are tested as individual substances, while in GE*RM*-Vet and human clinical isolates frequently a combination of a sulfonamide and trimethoprim is tested. Colistin and carbapenems have also not been taken into consideration. Colistin is used as a last resort antibiotic in the human sector. However, for methodological reasons phenotypical resistance data to colistin generated with automated methods are not considered reliable. Regarding carbapenems, different substances were used for animal clinical (imipenem) and non-clinical isolates (meropenem) and therefore data were not considered comparable. Moreover, resistance to carbapenem is extremely rare in animals [69] and also rare in humans in Germany [70].

We used SIR results based on clinical evaluation criteria for humans from EUCAST, as we could re-evaluate the quantitative data from the animal monitoring systems based on these breakpoints. As for the human data, either no quantitative data were available or the tested range was so narrow that a re-evaluation according to ECOFFs was not possible.

This study highlights substantial differences between the three monitoring and surveillance systems (Table 1). Differences in data collection (surveillance versus monitoring), participation system (mandatory versus voluntarily), observed populations (humans versus different animal populations), AST (panel, methods and results) and evaluation criteria (clinical breakpoints and epidemiological cut-off values) should be carefully considered for comparative analysis. For the purpose of comparing resistance proportions, it would be desirable that the One Health community strives towards harmonized evaluation criteria for each antimicrobial in isolates from humans, food-producing animals and food. Alternatively, quantitative data, such as MIC values, need to be collected for allowing the interpretation using different standards based on any required analysis. Rational criteria should be shaped based on various purposes, such as for treatment decisions and comparative analysis of different resistant proportions across different sectors. Joint harmonized MIC value ranges for comparative analyses of human and animal data would better fit for the analysis.

Since routine standardized diagnostics differ between human and animal sectors, it needs to be investigated whether the different laboratory methods yield comparable results. Routine methods are always a compromise between scientific accuracy and economic needs. Increasing costs might discourage widespread use of costly and laborious AST methods in routine laboratories, an aspect that is less relevant in monitoring programs with limited numbers of isolates.

To the best of our knowledge, this is the first study that systematically compares the routine laboratory surveillance and monitoring systems for AR in humans with different animal populations and food of animal origin in Germany using cluster analysis. Within the limitations noted above, our results indicate that patterns of resistance combinations are able to provide insights in similarities and discrepancies between isolates from different human and animal populations. Given the current situation on surveillance and monitoring for AR in Germany, we considered it the best approach to compare the national data on AR in *E*. *coli* from humans, different animal populations and food based on their phenotypical resistance combinations. Regional analyses within the country and across countries might provide valuable additional insights. However, further stratification of the data would lead to very small strata for some of the populations. This would likely lead to exclusion of several populations from the analysis. In this study, we would like to avoid this type of exclusion to be able to validly compare as many populations as possible. Although phenotypic datasets are able to promote the study on resistance combinations, the findings of this study suggest a number of directions, which future studies on molecular level on AR might profitably take. Integration of whole genome sequencing (WGS) into surveillance might help further research into resistance genes similarities. Initiatives on implementation of WGS in AR monitoring system for animals have been already started [71, 72]. As genomic information provides better insights into resistance mechanisms, mobile genetic elements, chromosomal mutations and intrinsic resistance, its inclusion in the comparative analysis should be further promoted.

## Conclusion

This study provides insights into possible analyses of AR phenotypical data from routine surveillance and monitoring in Germany. Despite differences in collected variables within the different surveillance and monitoring systems, cluster analysis has shown similarities and discrepancies between resistance patterns in isolates from humans and different animal populations for four frequently tested antibiotics. Using our datasets and analytical approach, we are not able to substantiate any transmission between humans, animals and foods. However, if the observed populations clustered separately, it is unlikely that a substantial amount of transmission between the populations has occurred. Initiatives built based on these results might promote successful ‘One Health’ improvements across human and different animal populations in Germany.

## Supporting information

S1 Table Fixed antibiotic panel from *Zoonosis-Monitoring* for non-clinical (commensal)—and from GE*RM*-Vet for clinical (pathogen)—*E*. *coli*. (DOCX)

S2 Table EUCAST clinical breakpoints for humans (resistant). (DOCX)

S3 Table Details on all clinical samples from ARS systems along with absolute numbers and percentages. (DOCX)

S4 Table Raw data from 41 different human and animal populations from 2014 to 2017 in Germany. (DOCX)

S1 Fig Determination of clusters. Results of the A) elbow method and B) silhouette plot to determine and confirm the optimum number of clusters.
(DOCX)

S2 Fig Sensitivity analysis. Results of cluster analysis considering A) only cefotaxime, ciprofloxacin and gentamicin (i.e. without ampicillin), B) only ampicillin, ciprofloxacin and gentamicin (i.e. without cefotaxime), C) only ampicillin, cefotaxime and gentamicin (i.e. without gentamicin) and D) only ampicillin, cefotaxime and ciprofloxacin (i.e. without gentamicin).
(DOCX)
