# Supplementary material for: Cluster analysis of resistance combinations in Escherichia coli from different human and animal populations in Germany 2014-2017
Source: PLoS One. 2021 Jan 20;16(1):e0244413. doi: 10.1371/journal.pone.0244413 (PMC7817003; doi:10.1371/journal.pone.0244413)
Supplement: S1 Table — (DOCX) [file pone.0244413.s001.docx]

**S1 Table. Fixed antibiotic panel from *Zoonosis-Monitoring* for non-clinical (commensal) - and from GE*RM*-Vet for clinical (pathogen) - *E. coli***

| **Antibiotic Groups** | **Antibiotic Name from ZoMo** | **Antibiotic Name from GE*RM*-Vet** |
| --- | --- | --- |
| Aminoglycosides | Gentamicin | Gentamicin |
|  |  | Neomycin |
|  |  | Streptomycin |
| Carbapenems | Meropenem | Imipenem |
| Cephalosphorins | Cefotaxime | Cefoperazon |
|  | Ceftazidime | Cefotaxime |
|  |  | Cefquinom |
|  |  | Ceftiofur |
| Folic-Acid Inhibitors | Trimethophrim | Trimetophrim/Sulfamethoxazole |
|  | Sulfamethoxazole |  |
| Macrolides | Azithromycin | Tilmicosin |
|  |  | Tulathromycin |
|  |  | Spiramycin |
| Penicillins | Ampicillin | Amoxciliin/Clavulan acid |
|  |  | Ampicillin |
|  |  | Penicillin G |
| Phenicoles | Chloramphenicole | Florfenicol |
| Pleuromutiline |  | Tiamulin |
| Polymxyins | Colistin | Colistin |
| Quinolones | Nalidixic Acid | Nalidixic Acid |
|  | Ciprofloxacin | Ciprofloxacin |
|  |  | Enrofloxacin |
|  |  | Marbofloxacin |
| Tetracyclines | Tetracycline | Tetracycline |
|  |  | Doxyclcine |
| Tigecycline | Tigecycline |  |
