# Supplementary material for: Cluster analysis of resistance combinations in Escherichia coli from different human and animal populations in Germany 2014-2017
Source: PLoS One. 2021 Jan 20;16(1):e0244413. doi: 10.1371/journal.pone.0244413 (PMC7817003; doi:10.1371/journal.pone.0244413)
Supplement: S2 Table — (DOCX) [file pone.0244413.s002.docx]

**S2 Table**. **EUCAST clinical breakpoints for humans (resistant)**

| **Antibiotics** | **MIC** |
| --- | --- |
| Ampicillin (AMP) | > 8 µg/µl |
| Cefotaxime (CTX) | > 2 µg/µl |
| Ciprofloxacin (CIP) | > 0.5 µg/µl |
| Gentamicin (GEN) | > 4 µg/µl |
