# Supplementary material for: Cluster analysis of resistance combinations in Escherichia coli from different human and animal populations in Germany 2014-2017
Source: PLoS One. 2021 Jan 20;16(1):e0244413. doi: 10.1371/journal.pone.0244413 (PMC7817003; doi:10.1371/journal.pone.0244413)
Supplement: S3 Table — (DOCX) [file pone.0244413.s003.docx]

**S3 Table. Details on all clinical samples from ARS systems along with absolute numbers and percentages**

| **Material groups (n = 324,304)** | **Comments** | **Absolute numbers (%)** |
| --- | --- | --- |
| Swabs | eyes, nose, tonsils/throat, intraoperative swabs, urogenital, tongue, others and non-specified swabs | 48,506 (15.0%) |
| Blood | blood | 19,361 (6.0%) |
| Punctate | biopsies from tissues, liquor, pleural cavity, abscess, ascites, joint, others and non-specified punctate | 6,513 (2.0%) |
| Respiratory tract samples | bronchial lavage, bronchial secretions, sputum, tracheal secretion, and other respiratory samples | 13,709 (4.2) |
| Wound samples | swabs abscess and wound swab | 22,999 (7.1%) |
| Urine | urine | **210,005 (64.8%)** |
| Other Samples | ejaculate, catheters, faeces | 3,211 (1.0%) |
