# Supplementary material for: Cluster analysis of resistance combinations in Escherichia coli from different human and animal populations in Germany 2014-2017
Source: PLoS One. 2021 Jan 20;16(1):e0244413. doi: 10.1371/journal.pone.0244413 (PMC7817003; doi:10.1371/journal.pone.0244413)
Supplement: S4 Table — (DOCX) [file pone.0244413.s004.docx]

S4 Table. Raw data from 41 different human and animal populations from 2014 to 2017 in Germany.

| **Populations** | **No. of isolates (n = 333,496)** | **Susceptible to all antibiotics (0000)** | **Resistant to AMP only (1000)** | **Resistant to CTX only (0100)** | **Resistant to CIP only (0010)** | **Resistant to GEN only (0001)** | **Resistant to AMP and CTX (1100)** | **Resistant to AMP and CIP (1010)** | **Resistant to AMP and GEN (1001)** | **Resistant to CTX and CIP (0110)** | **Resistant to CTX and GEN (0101)** | **Resistant to CIP and GEN (0011)** | **Resistant to AMP, CTX and CIP (1110)** | **Resistant to AMP, CTX and GEN (1101)** | **Resistant to AMP, CIP and GEN (1011)** | **Resistant to CTX, CIP and GEN (0111)** | **Resistant to all antibiotics (1111)** |
| --- | --- | --- | --- | --- | --- | --- | --- | --- | --- | --- | --- | --- | --- | --- | --- | --- | --- |
| Bivalves, F | 42 | 38 | 3 | 0 | 0 | 0 | 0 | 0 | 1 | 0 | 0 | 0 | 0 | 0 | 0 | 0 | 0 |
| Bivalves, R | 58 | 51 | 5 | 0 | 2 | 0 | 0 | 0 | 0 | 0 | 0 | 0 | 0 | 0 | 0 | 0 | 0 |
| Bovine Mastitis, C | 378 | 307 | 33 | 0 | 0 | 1 | 12 | 7 | 1 | 0 | 0 | 0 | 6 | 1 | 2 | 0 | 8 |
| Bovine Meat, R | 115 | 102 | 9 | 0 | 0 | 0 | 3 | 1 | 0 | 0 | 0 | 0 | 0 | 0 | 0 | 0 | 0 |
| Bovine Milk Conv, F | 122 | 115 | 6 | 0 | 0 | 0 | 0 | 1 | 0 | 0 | 0 | 0 | 0 | 0 | 0 | 0 | 0 |
| Bovine Milk Org, F | 74 | 73 | 1 | 0 | 0 | 0 | 0 | 0 | 0 | 0 | 0 | 0 | 0 | 0 | 0 | 0 | 0 |
| Bovines < 1 year, S | 433 | 282 | 128 | 0 | 2 | 1 | 7 | 9 | 3 | 0 | 0 | 0 | 1 | 0 | 0 | 0 | 0 |
| Bovines <1 year, C | 534 | 140 | 112 | 1 | 7 | 3 | 30 | 45 | 28 | 0 | 0 | 2 | 42 | 28 | 34 | 0 | 62 |
| Breeder Chickens, F | 56 | 39 | 10 | 0 | 2 | 1 | 0 | 2 | 2 | 0 | 0 | 0 | 0 | 0 | 0 | 0 | 0 |
| Broiler Meat, R | 363 | 148 | 127 | 0 | 13 | 2 | 12 | 49 | 4 | 0 | 0 | 1 | 3 | 1 | 3 | 0 | 0 |
| Broilers Conv, F | 299 | 83 | 155 | 0 | 5 | 1 | 2 | 51 | 1 | 0 | 0 | 0 | 0 | 1 | 0 | 0 | 0 |
| Broilers Org, F | 31 | 22 | 5 | 0 | 1 | 1 | 0 | 2 | 0 | 0 | 0 | 0 | 0 | 0 | 0 | 0 | 0 |
| Broilers, C | 232 | 141 | 64 | 1 | 4 | 4 | 3 | 8 | 2 | 0 | 0 | 0 | 0 | 5 | 0 | 0 | 0 |
| Broilers, F | 184 | 44 | 93 | 0 | 7 | 0 | 3 | 34 | 2 | 0 | 0 | 0 | 1 | 0 | 0 | 0 | 0 |
| Broilers, S | 404 | 149 | 188 | 0 | 12 | 12 | 1 | 28 | 11 | 0 | 0 | 0 | 0 | 0 | 3 | 0 | 0 |
| Cattle, C | 193 | 100 | 35 | 0 | 2 | 0 | 6 | 14 | 5 | 0 | 0 | 0 | 7 | 3 | 9 | 0 | 12 |
| Fattening Pigs, S | 439 | 299 | 123 | 0 | 3 | 0 | 6 | 2 | 1 | 0 | 0 | 0 | 4 | 1 | 0 | 0 | 0 |
| Growers <50 kg, F | 210 | 137 | 63 | 0 | 0 | 0 | 1 | 4 | 4 | 0 | 0 | 0 | 1 | 0 | 0 | 0 | 0 |
| Growers, C | 129 | 44 | 70 | 0 | 2 | 1 | 6 | 1 | 2 | 0 | 0 | 0 | 0 | 3 | 0 | 0 | 0 |
| Humans, A | 96455 | 52294 | 24913 | 2 | 2611 | 208 | 2225 | 6186 | 1210 | 1 | 0 | 100 | 3435 | 343 | 1367 | 2 | 1558 |
| Humans, Gw | 197521 | 94911 | 54253 | 6 | 5153 | 349 | 6754 | 14516 | 2320 | 3 | 1 | 243 | 10538 | 1046 | 3178 | 0 | 4250 |
| Humans, ICU | 30328 | 12949 | 8805 | 0 | 662 | 37 | 1435 | 2237 | 349 | 2 | 0 | 35 | 2188 | 259 | 510 | 0 | 860 |
| Laying Hens, C | 557 | 461 | 72 | 0 | 2 | 5 | 3 | 8 | 3 | 0 | 0 | 1 | 1 | 0 | 0 | 1 | 0 |
| Laying Hens, F | 347 | 300 | 30 | 0 | 1 | 0 | 9 | 3 | 1 | 0 | 0 | 0 | 1 | 0 | 1 | 0 | 1 |
| Piglets, C | 417 | 147 | 195 | 0 | 8 | 4 | 19 | 17 | 15 | 0 | 0 | 2 | 1 | 0 | 2 | 0 | 7 |
| Pigs, C | 346 | 165 | 131 | 1 | 2 | 5 | 14 | 16 | 7 | 0 | 1 | 0 | 1 | 1 | 2 | 0 | 0 |
| Pork, R | 155 | 116 | 34 | 0 | 0 | 0 | 4 | 0 | 1 | 0 | 0 | 0 | 0 | 0 | 0 | 0 | 0 |
| Raw Sausages, R | 69 | 55 | 8 | 0 | 0 | 0 | 1 | 4 | 0 | 0 | 0 | 0 | 0 | 0 | 1 | 0 | 0 |
| Roe Deer Hunted, W | 269 | 265 | 3 | 0 | 0 | 0 | 1 | 0 | 0 | 0 | 0 | 0 | 0 | 0 | 0 | 0 | 0 |
| Shrimps, R | 20 | 15 | 1 | 0 | 1 | 0 | 2 | 0 | 0 | 0 | 0 | 0 | 0 | 0 | 1 | 0 | 0 |
| Small Animals, C | 312 | 199 | 44 | 1 | 4 | 1 | 10 | 25 | 3 | 0 | 0 | 0 | 11 | 3 | 4 | 0 | 7 |
| Sows, C | 24 | 17 | 6 | 0 | 0 | 0 | 0 | 0 | 0 | 0 | 0 | 0 | 0 | 0 | 1 | 0 | 0 |
| Sows, F | 272 | 197 | 63 | 1 | 1 | 1 | 1 | 3 | 3 | 0 | 0 | 0 | 0 | 1 | 0 | 0 | 1 |
| Table Eggs, R | 90 | 79 | 9 | 0 | 0 | 1 | 0 | 1 | 0 | 0 | 0 | 0 | 0 | 0 | 0 | 0 | 0 |
| Turkey Meat, R | 356 | 107 | 152 | 0 | 6 | 3 | 3 | 50 | 15 | 0 | 0 | 0 | 7 | 0 | 11 | 0 | 2 |
| Turkeys, C | 327 | 190 | 106 | 0 | 2 | 3 | 0 | 14 | 10 | 0 | 0 | 0 | 1 | 0 | 1 | 0 | 0 |
| Turkeys, F | 346 | 119 | 131 | 0 | 5 | 5 | 0 | 60 | 11 | 0 | 0 | 1 | 2 | 0 | 12 | 0 | 0 |
| Turkeys, S | 372 | 126 | 154 | 0 | 5 | 3 | 0 | 54 | 16 | 0 | 0 | 1 | 3 | 0 | 7 | 0 | 3 |
| Venisons, R | 150 | 147 | 3 | 0 | 0 | 0 | 0 | 0 | 0 | 0 | 0 | 0 | 0 | 0 | 0 | 0 | 0 |
| Weaners, F | 250 | 123 | 105 | 0 | 0 | 0 | 8 | 6 | 4 | 0 | 0 | 0 | 2 | 0 | 1 | 0 | 1 |
| Wild Boar Hunted, W | 217 | 215 | 0 | 0 | 1 | 0 | 0 | 1 | 0 | 0 | 0 | 0 | 0 | 0 | 0 | 0 | 0 |
