# Supplementary material for: Cluster analysis of resistance combinations in Escherichia coli from different human and animal populations in Germany 2014-2017
Source: PLoS One. 2021 Jan 20;16(1):e0244413. doi: 10.1371/journal.pone.0244413 (PMC7817003; doi:10.1371/journal.pone.0244413)

**S1 Fig. Determination of Clusters.** Results of the A) elbow method and B) silhouette plot to determine and confirm the optimum number of clusters.

A.


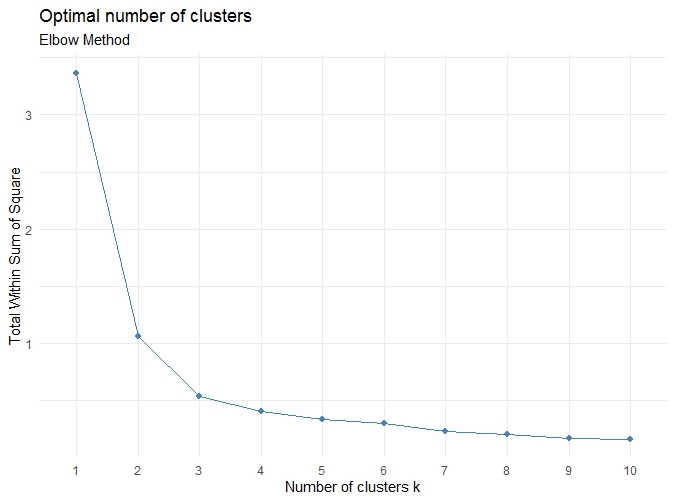


B.


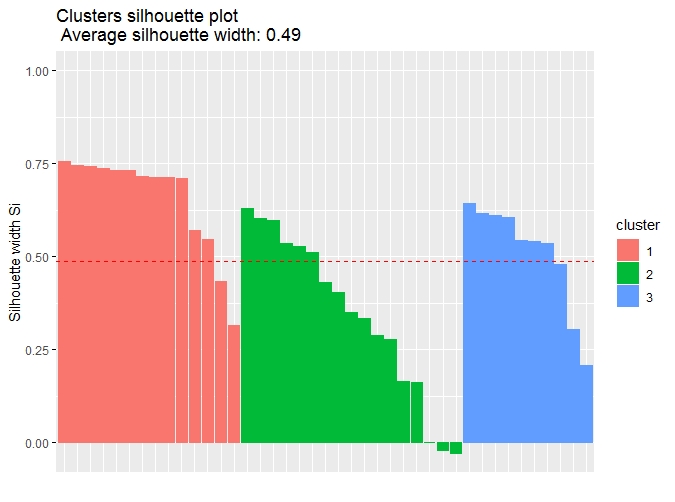

Supplement: S1 Fig — Results of the A) elbow method and B) silhouette plot to determine and confirm the optimum number of clusters. (DOCX) [file pone.0244413.s005.docx]
