# Supplementary material for: Cluster analysis of resistance combinations in Escherichia coli from different human and animal populations in Germany 2014-2017
Source: PLoS One. 2021 Jan 20;16(1):e0244413. doi: 10.1371/journal.pone.0244413 (PMC7817003; doi:10.1371/journal.pone.0244413)
Supplement: S2 Fig — Results of cluster analysis considering A) only cefotaxime, ciprofloxacin and gentamicin (i.e. without ampicillin), B) only ampicillin, ciprofloxacin and gentamicin (i.e. without cefotaxime), C) only ampicillin, cefotaxime and gentamicin (i.e. without gentamicin) and D) only ampicillin, cefotaxime and ciprofloxacin (i.e. without gentamicin). (DOCX) [file pone.0244413.s006.docx]

**S2 Fig. Sensitivity Analysis.** Results of cluster analysis considering A) only cefotaxime, ciprofloxacin and gentamicin (i.e. without ampicillin), B) only ampicillin, ciprofloxacin and gentamicin (i.e. without cefotaxime), C) only ampicillin, cefotaxime and gentamicin (i.e. without gentamicin) and D) only ampicillin, cefotaxime and ciprofloxacin (i.e. without gentamicin).

A.


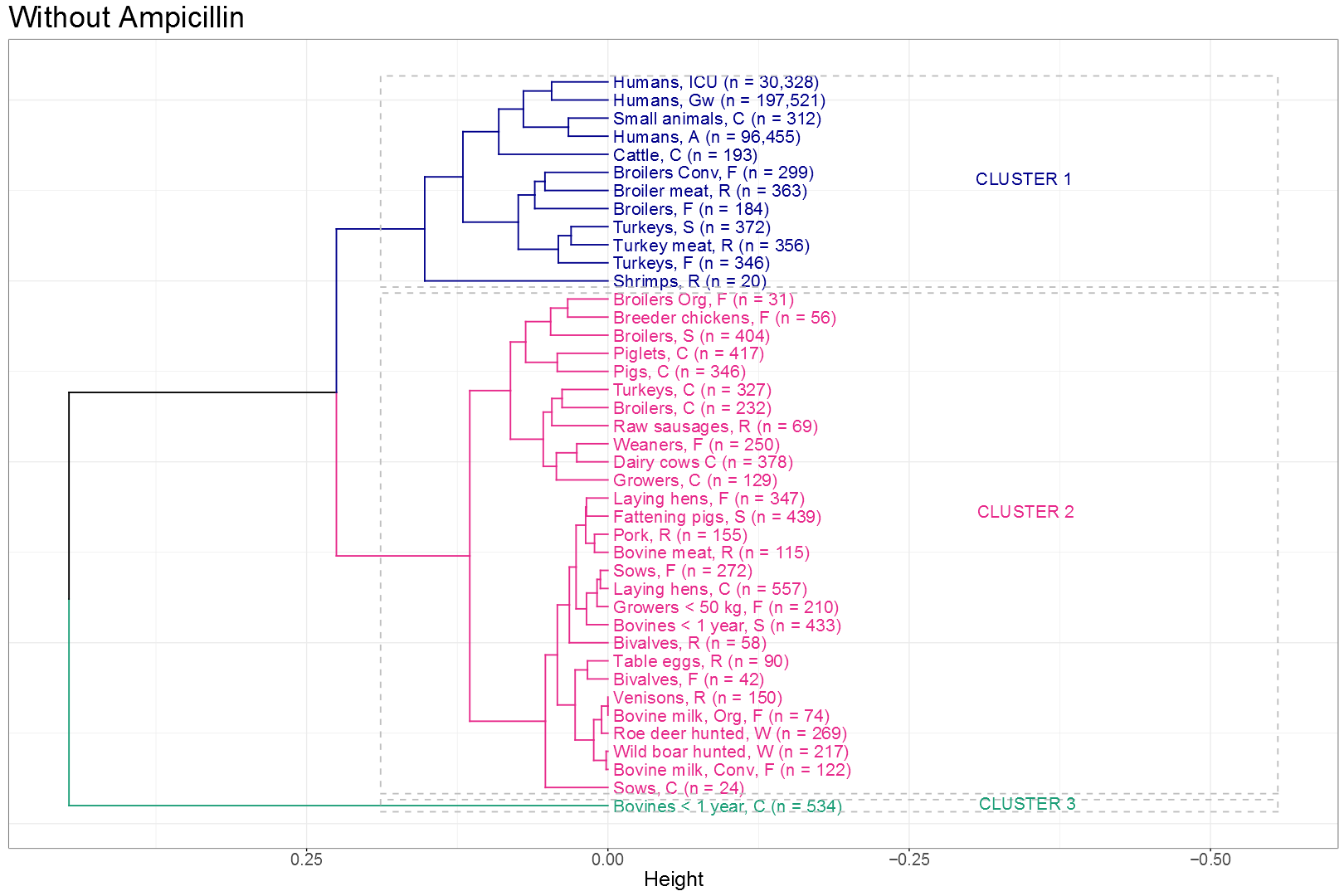


B.
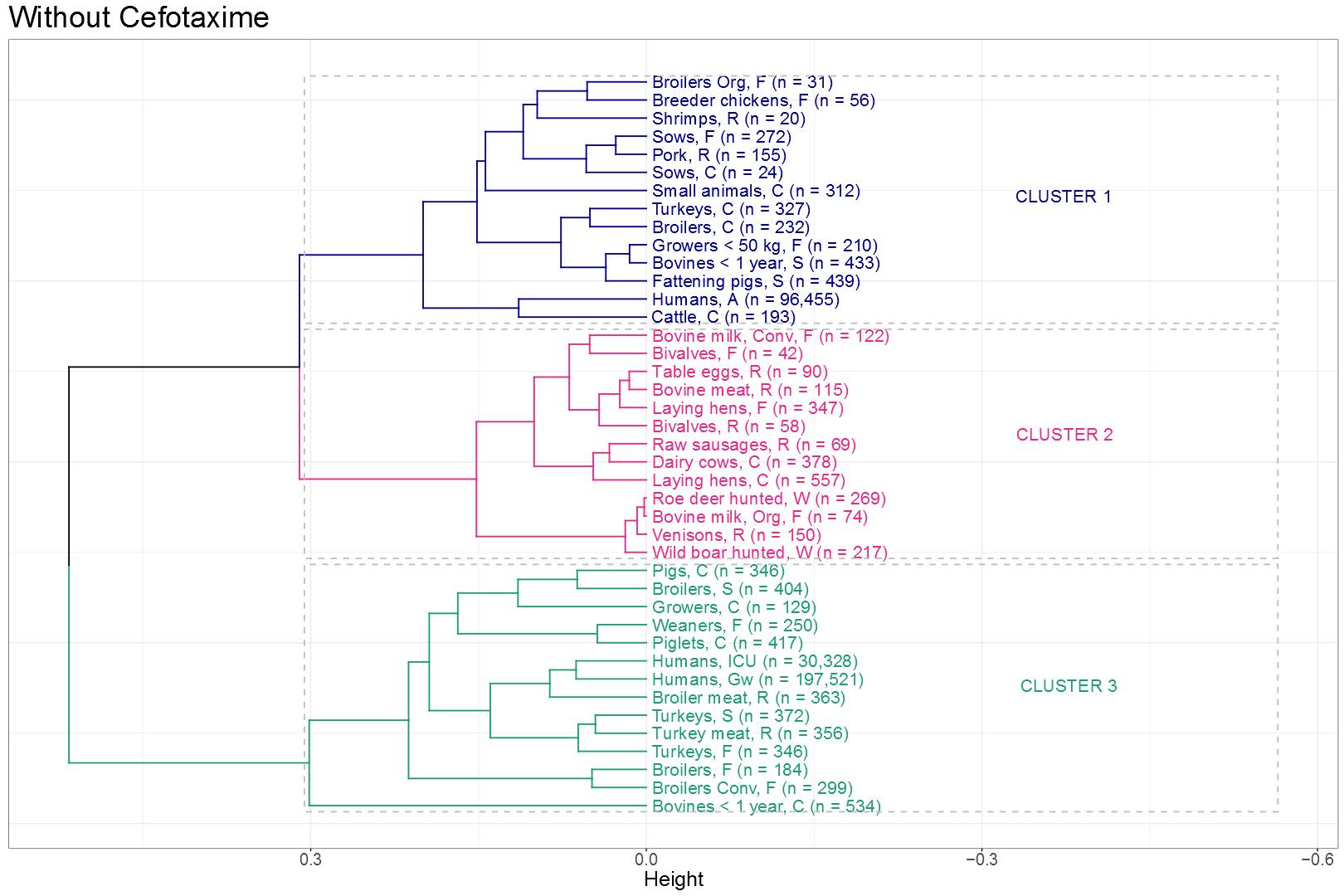


C.
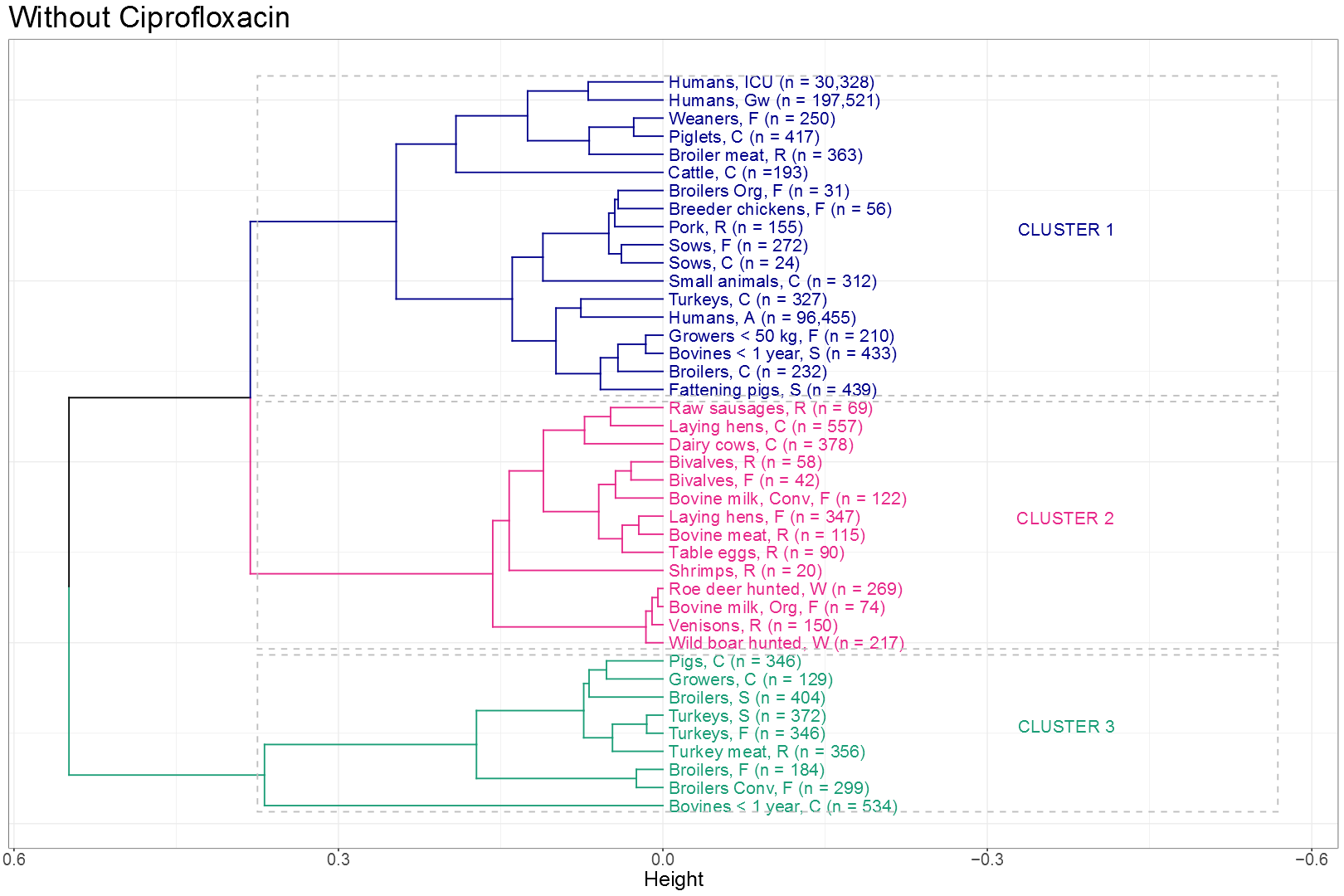


D. **
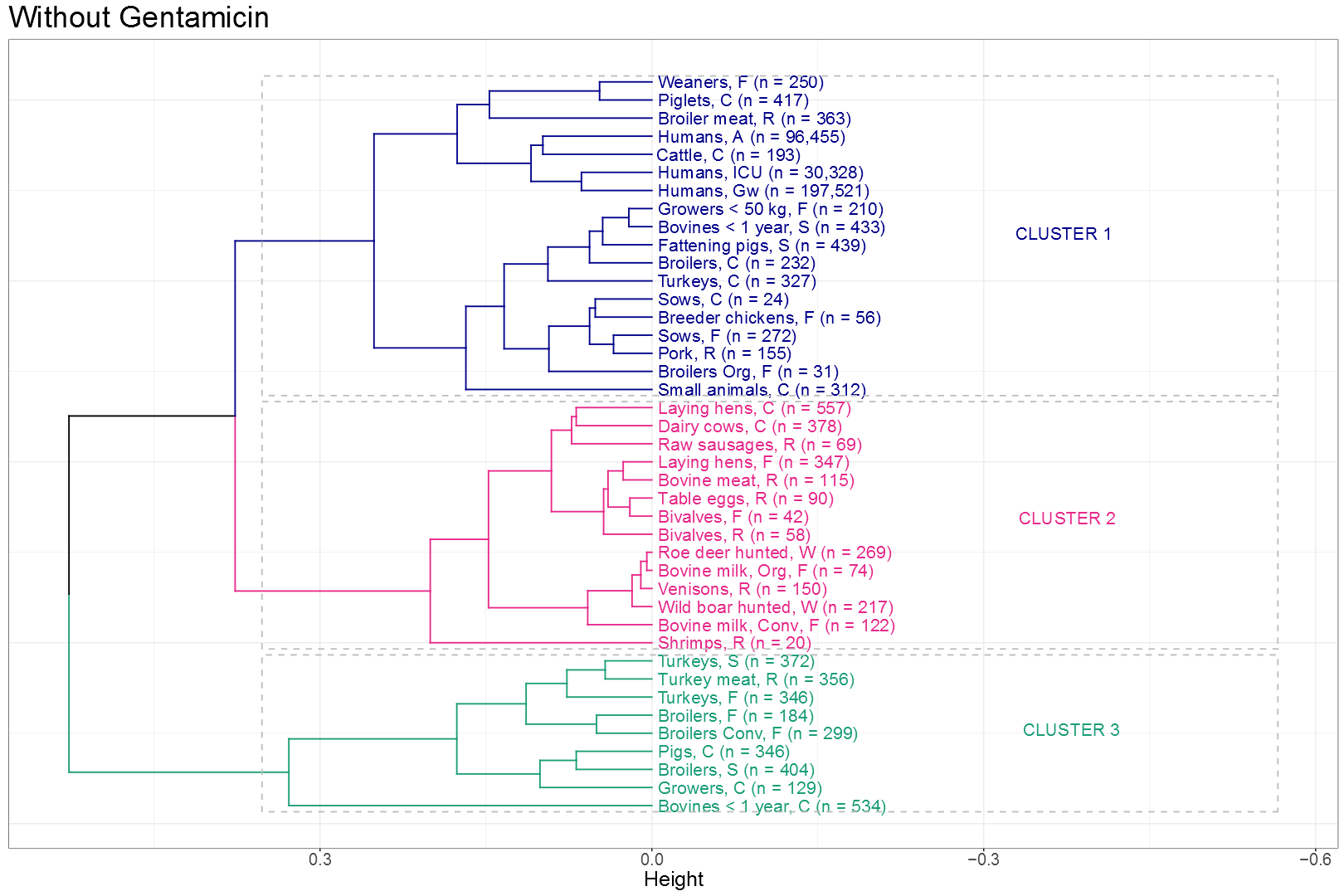
**
